# Cryptic Extensibility in von Willebrand Factor Revealed by Molecular Nanodissection

**DOI:** 10.3390/ijms25137296

**Published:** 2024-07-02

**Authors:** Mária Csilla Csányi, Dominik Sziklai, Tímea Feller, Jolán Hársfalvi, Miklós Kellermayer

**Affiliations:** 1 Institute of Biophysics and Radiation Biology, Semmelweis University, Tűzoltó u. 37-47, H1094 Budapest, Hungary; csanyi.maria@semmelweis.hu (M.C.C.);; 2 Discovery and Translational Science Department, Leeds Institute of Cardiovascular and Metabolic Medicine, University of Leeds, Leeds LS29JT, UK; 3 HUNREN-SE Biophysical Virology Group, Tűzoltó Str. 37-47, H1094 Budapest, Hungary

**Keywords:** single-molecule mechanics, receding meniscus, mechanosensitive domains, nanomanipulation, atomic force microscopy

## Abstract

Von Willebrand factor (VWF) is a multimer with a variable number of protomers, each of which is a head-to-head dimer of two multi-domain monomers. VWF responds to shear through the unfolding and extension of distinct domains, thereby mediating platelet adhesion and aggregation to the injured blood vessel wall. VWF's C_1-6_ segment uncoils and then the A_2_ domain unfolds and extends in a hierarchical and sequential manner. However, it is unclear whether there is any reservoir of further extensibility. Here, we explored the presence of cryptic extensibility in VWF by nanodissecting individual, pre-stretched multimers with atomic force microscopy (AFM). The AFM cantilever tip was pressed into the surface and moved in a direction perpendicular to the VWF axis. It was possible to pull out protein loops from VWF, which resulted in a mean contour length gain of 217 nm. In some cases, the loop became cleaved, and a gap was present along the contour. Frequently, small nodules appeared in the loops, indicating that parts of the nanodissected VWF segment remained folded. After analyzing the nodal structure, we conclude that the cryptic extensibility lies within the C_1-6_ and A_1-3_ regions. Cryptic extensibility may play a role in maintaining VWF’s functionality in extreme shear conditions.

## 1. Introduction

**Figure 1:**
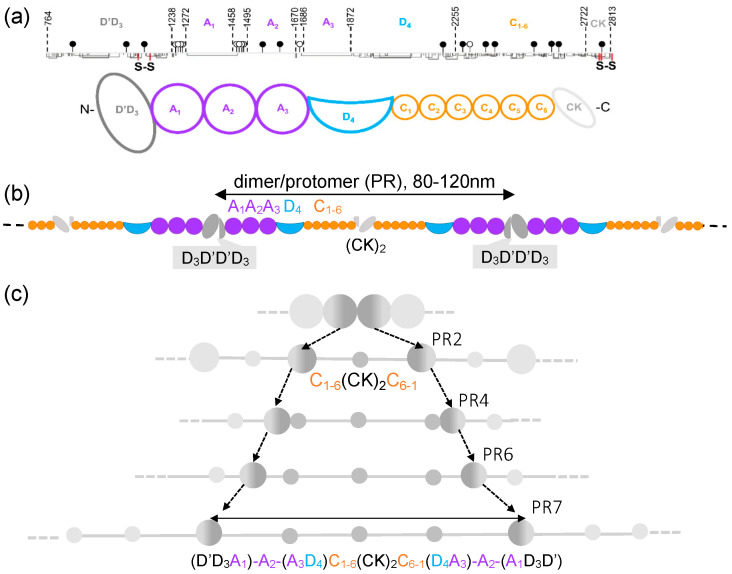
Structure and hierarchical extension of VWF. (**a**) Domain structure of VWF monomer. Domains with similar structure have the same color. The numbers in the top line indicate the number of amino acids at the domain boundaries; the closed and open lollipops mark the locations of the N- and O-linked oligosaccharides, respectively. Lines pointing downward are cysteines, and connected ones are disulfides. The two S-S labels are N-N and C-C terminal intermonomer disulfide bonds [8]. (**b**) Domain structure of VWF multimer. The two-headed arrow indicates one protomer. It is not known whether domains other than A_2_ are also extensible. (**c**) Schematics of VWF protomer elongation via structural intermediates observed with AFM [5]. The dashed arrows indicate the progressive change in the protomer boundary through the gradually extended protomer classes (PR2 to PR7). During extension, small nodules separated by rod-like structures emerge. The double-headed arrow indicates the pre-stretched protomer.

Von Willebrand factor (VWF) is a multimeric glycoprotein that circulates in the blood as a random coil with hidden functional epitopes. VWF mediates platelet adhesion and aggregation at the injured vessel wall by exposing epitopes to the platelets’ GPIb-α and integrin αIIbβ3 receptors upon extension by shear stress [1]. Exposure to stress leads to the elongation of specific domains, revealing molecular sites for self-association or proteolytic cleavage and the for further enhancement of platelet adhesion and activation [2,3]. The mature VWF monomer is a 2050-residue protein (molecular weight/MW 250 kDa) containing 12 N- and 10 O-linked oligosaccharide chains (Figure 1a). It displays an abundance of cysteines that constitute 8.3% of its amino acids, compared with the average 2.3% observed in human proteins. The monomer is structurally partitioned into distinct domains, usually represented as D’D3-A1-A2-A3-D4-C1-C2-C3-C4-C5-C6-CK, which are connected by flexible hinge regions and many of which are stabilized by disulfide bonds [4]. For ease of reference, here, we use a subscripted domain labeling D’D_3_A_1_A_2_A_3_D_4_C_1-6_CK (Figure 1a) [5]. The structurally relevant smallest unit of VWF is the protomer, which is a head-to-head dimer of two monomers connected with disulfides between their CK domains that displays two N-terminals at both ends (Figure 1b). Multimerization, up to one hundred fold physiologically, occurs by disulfide bridge formation between the N-terminal D’D_3_ domains of consecutive protomers [4,6,7].

As identified in early EM images, elongated protomers have a symmetric structure with two large nodules at both ends and a small nodule in the center, which are connected by flexible filaments or rods. The N- and C-terminals of the monomer have been assigned to large and small nodules, respectively [9]. Atomic force microscopy (AFM), due to its versatility and high topographical resolution, has been extensively used to characterize the nodules and rods of the VWF multimers under various experimental conditions [5,10,11,12]. Recently, we have shown, by analyzing human blood-plasma-derived VWF stretched with molecular combing, that protomers extend through structural intermediates, which can be grouped into seven distinct topographical classes (PR1–PR7). The most extended protomer is PR7, which is a linkage of N-terminal large nodule–thin segment–small nodule–thin segment–central small nodule–thin segment–small nodule–thin segment–N-terminal large nodule and corresponds to _N_D’D_3_A_1_A_2_A_3_D_4_C_1-6_(CK)_2_C_6-1_D_4_A_3_A_2_A_1_D_3_D'_N_. The schematics of the evolution of PR7 from upon protomer extension are shown in Figure 1c [5]. Positively charged regions of VWF tether the multimer extended by molecular combing to the mica surface, likely in a similar way to the binding of VWF multimers to GPIbα and heparin with the electropositive face of the A_1_ domains [13]. It remains to be explored whether additional domains or regions of VWF may be extended upon exposure to even greater or to spatially localized forces. AFM also allows for the direct nanomanipulation of surface-bound molecular structures, thereby revealing further structural and mechanical features of the investigated system. Such nanodissection has been employed to explore plasmid DNA [14], fibrous proteins [15,16,17], the giant muscle protein titin [18] and various other molecular structures [19] and may be utilized to inflict localized mechanical forces on VWF.

In the present work, we carried out molecular nanodissection on pre-stretched VWF multimers so as to reveal hidden cryptic extensibility. Our results suggest that such cryptic extensibility prevails in VWF and is associated with the A_1–3_ and C_1–6_ domains.

## 2. Results and Discussion

**Figure 2:**
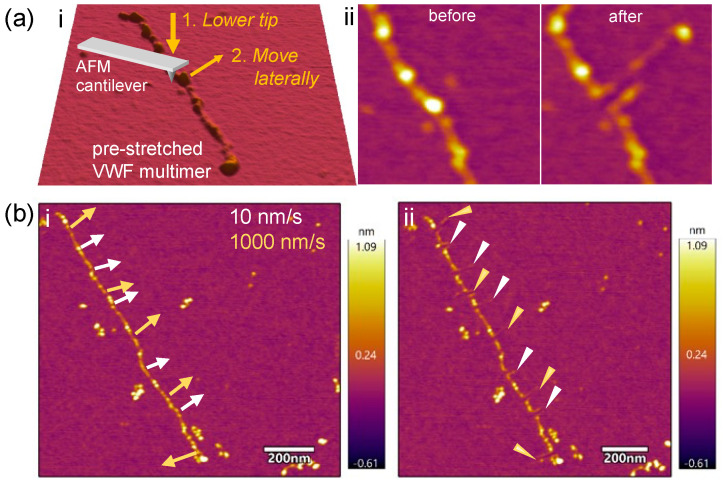
(**a**) VWF nanodissection with AFM. (i) Schematics of the procedure. (ii) Effect of nanodissection on VWF structure. The arrowhead points to the location where the AFM tip was initially lowered. The arrow indicates the direction and amplitude of tip motion. (**b**) Effect of cantilever speed on nanodissection. Height-contrast AFM images prior to (i) and following (ii) nanodissection with 10 (white arrows) and 1000 nm/s (yellow arrows), respectively. The arrows indicate the direction and the amplitude of the tip motion on (i), and the elongated structure on (ii).

**Figure 3:**
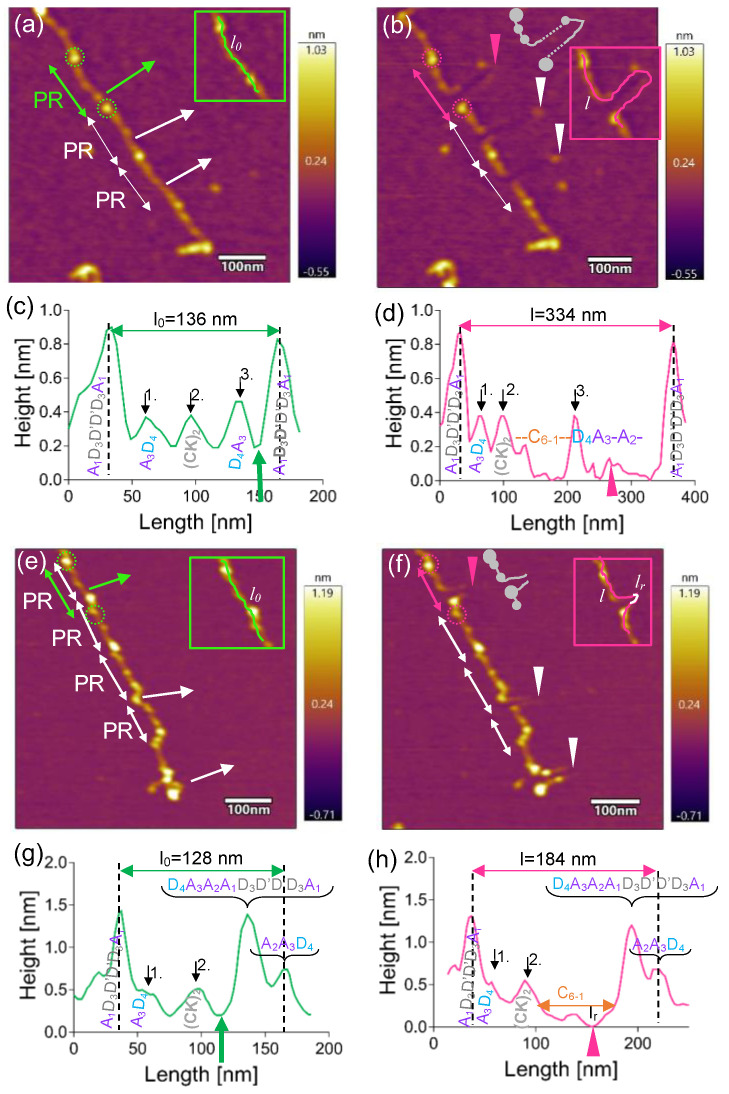
Nanodissection of VWF multimer pre-stretched with meniscus force. (**a**) VWF prior to and (**b**) following nanodissection, in which strand continuity was preserved. Double-headed arrows indicate protomers. The corresponding axial topographical height profiles are shown in (**c**,**d**), respectively. (**e**) VWF prior to and (**f**) following nanodissection, in which strand rupture occurred. The corresponding axial topographical height profiles are shown in (**g**,**h**), respectively. Green and magenta double-headed arrows indicate the analyzed VWF sections prior to and following nanodissection, respectively. The detailed analysis of the sections indicated with the white arrows is not shown here. The terminal nodules of the protomers that remained in place after nanodissection are encircled. Arrows perpendicular to the VWF multimer axis highlight the path of the AFM tip during the nanodissection procedure. Arrowheads point to the tips of the loops. The grey cartoons in (**b**,**f**) illustrate the schematic structures of the VWF strands following nanodissection. The black arrows on the topographical height profiles point to the small nodules. Abbreviations: *l_0_*, original length of the section (green line in inset); *l*, length after nanodissection (pink line in inset); *l_r_*, length of the ruptured section (white line in inset).

We investigated whether there was cryptic extensibility in VWF by nanodissecting individual pre-stretched multimers with AFM-based nanolithographic methods (Figure 2). In this process, we lowered the AFM cantilever tip onto the sample surface at an initial position determined based on the AFM image and exerted a force ranging between 150 and 600 nN (Figure 2a.i). The initial position was chosen to be vicinal to the VWF domain or segment to be manipulated. Subsequently, the cantilever tip was moved in a direction approximately perpendicular to the axis of the VWF multimer to a pre-determined amplitude, while the force was kept constant. As a result, a hairpin-like loop could be pulled out of the VWF multimer, which became stabilized by adsorption to the mica surface (Figure 2a.ii). Subsequent AFM imaging allowed us to quantitate the features of the protein loop. A few initial observations can already be made: (1) the global structure and arrangement of the mother VWF multimer, from which the loop has been pulled out, is essentially unaltered, indicating that it is firmly adsorbed on the surface [16,18]; (2) the upper and lower strands of the loop run essentially in parallel, suggesting that the protein loop's proximal and distal attachment points are bound to the mica surface sufficiently strongly so that only the VWF part directly dislodged by the AFM tip becomes pulled; (3) the surface of the loop is uneven, and nodules may appear along its contour; (4) thinning may be observed along the loop, suggesting that complete protein unfolding took place [20]; and (5) discontinuities may be observed at the tip of the loop, indicating that it became locally ruptured due to overstretching (see Figure 2b.ii and Figure 3f). To test whether the tip velocity influences loop elongation, we translated the AFM tip with different velocities (10, 100 and 1000 nm/s) [18]. The lengths of the loop strands were comparable regardless of the velocity (Figure 2b); therefore, we used the intermediate velocity of 100 nm/s in subsequent experiments.

### 2.1. Continuous VWF Loops Reveal Sources of Cryptic Extensibility

A section of a pre-stretched multimer containing three protomers in tandem is shown in Figure 3a. Each protomer was nanodissected at slightly different locations, either near the N-junction (top and middle) or at the (CK)_2_ knot (bottom). As a result, protein loops with amplitudes reaching ~100 nm emerged (Figure 3b). The top protomer (green double arrow) was analyzed further. This protomer originally contained two large terminal nodules and three small nodules in between, indicating that its structure corresponded to the PR6 intermediate (Figure 1c). Topographical profile analysis revealed that the initial protomer length (*l*_0_) was 136 nm (Figure 3c). The most plausible interpretation is that the two large terminal nodules correspond to the A_1_D_3_D’D’D_3_A_1_ domains and the three small ones to one central (CK)_2_ domain and two bounding A_3_D_4_ domains. The A_2_ domains connect the large nodules to the small ones, and the C_1-6_ domains connect the small nodules to the central nodule. However, these connections are not discernible, because the nodules are too close to each other. Following nanodissection (Figure 3b), the terminal large nodules (magenta dotted circles) bounding the VWF protomer (magenta double-headed arrow) remained clearly visible. While the entire contour of the loop is roughly discernible, the long sections in both the proximal and distal strands have thinned so much as to indicate extensive local domain unfolding. Furthermore, a small nodule appeared in the proximal strand of the loop. The originally 136-nm-long VWF section became extended by (Δ*l*) 198 nm, which amounts to a 2.5-fold relative extension (*E*) in this experiment. The thinned sections in the proximal and distal strands of the loop are 55 and 127 nm long and correspond most plausibly to the C_1-6_ and A_3_A_2_ domains, respectively (Figure 3d). The C_1-6_ is 1.2-fold longer than measured before [5,9,21], suggesting that either partial domain unfolding or linker region extension has occurred within this section. The distal thin segment is almost twice as long as the theoretical length of the unfolded A_2_ domain (73 nm) [5,22,23], indicating that the partial unfolding of a domain vicinal to the otherwise extensible A_2_ domain must be invoked. Considering that the topographical height of the distal terminal nodule, which contains the A_1_ domain, remained unchanged, we propose that it is the A_3_ domain that has gone through partial unfolding in this particular case. The statistical comparison of all analyzed nanodissected VWF loops revealed that the median length of the thinned segments was 76 nm (IQR 52-110 nm, *n* = 23) and 96 nm (IQR 64-121 nm, *n* = 22) in the A_2–3_ and C_1–6_ domain regions, respectively. Considering that the A_2–3_ and C_1–6_ domains represent the thinned and hence partially unfolded regions of the nanodissected VWF, the small nodule along the contour is the D_4_ domain. Thus, the A_2–3_ and C_1–6_ domains provide additional extensibility to VWF that may be recruited under mechanically demanding circumstances.

The exact mechanism of the additional extensibility in the VWF A and C domains remains a question. The C_1–6_ domains are stabilized by intra-domain disulfide crosslinks [4]. Recently, it has been shown that two disulfide bonds in the C_4_ domain can be partially reduced in human blood, and the resulting flexibility enhancement in the VWF dimer may increase platelet accessibility [24,25,26]. Among the VWF A domains, A_1_ and A_3_ are stabilized by long-range disulfide bonds that crosslink the C- and N-terminal ends in A_3_, but A_1_ has C- and N-terminal extensions past the disulfide bridge [27]. In A_3_, the disulfide bond directly resists force. By contrast, in A_1_, an extensive hydrogen bond network must be broken before the disulfide bridge becomes exposed to force [4,28]. Mechanical force might induce A_3_ elongation via loop opening [29], which is the likely scenario that took place during the nanodissection experiments shown here. In the A_2_ domain, there is a disulfide bridge within the N-terminal α-helix, but it does not contribute to the mechanical stability of the domain; therefore, it can be unfolded by ~20 pN force [11,30].

### 2.2. Ruptured VWF Loops Uncover Mechanically Weak Regions

In a fraction (27.5%) of the experiments, we observed complete discontinuity at the tip of the nanodissected VWF loop, indicating that the protein had become mechanically ruptured (Figure 3e–h). Figure 3e shows a VWF multimer in which four protomers in tandem can be discerned. The top protomer, the topographical structure of which was analyzed here in detail, has an initial end-to-end length (*l*_0_) of 128 nm, and its distal region corresponds to a tightly packed D_4_A_3_A_2_A_1_D_3_D’D’D_3_A_1_ followed by a half A_2_A_3_D_4_ small nodule (Figure 3g). Following the nanodissection of this region (Figure 3f), a loop is observed, at the tip of which a gap (with a gap width, *l*_r_, of 21 nm) can be identified. The total loop length (*l*), including the gap, is 184 nm, and, notably, no nodules can be identified along either the proximal or distal strands. Most plausibly, it was the C_6–1_ segment that became ruptured (Figure 3h), while the rest of the VWF domains remained in place. Among the eleven loops observed in our experimental set, four ruptures were located in the A_2_ domain and four in the C_1–6_ segment, suggesting that VWF's A- and C-domain regions not only harbor cryptic extensibility but they may be structurally vulnerable sites when exposed to excessive mechanical forces. Factors that influence protein chain rupture are the magnitude of shear stress, the adhesion forces between VWF epitopes and the substrate surface and the structural buffering capacity of the VWF domains. The system of hydrogen bonds and disulfide bridges along VWF may serve to both resist excessive forces and to provide a structural buffer under high shear. 

The statistics of the nanodissected VWF parameters are summarized in Table 1. The extension (Δ*l*) and relative extension (*E*) are significantly greater in the continuous loops than in the ruptured ones. By contrast, the topographical height parameters of the affected VWF segments are comparable. A possible explanation for this finding is that rupture might release the mechanical tension in the stretched loop, which then relaxes and elastically recoils. Furthermore, a fragment of the loop may adhere to the AFM cantilever tip and become removed altogether. Although the bulk statistics already point to the presence of hidden extensibility in VWF, such cryptic reservoirs can be further substantiated by comparing the maximum protomer length after nanosurgery with the maximum protomer length measured systematically in VWF multimers extended by shear only (i.e., maximum length of the PR7 conformer) [5]. Considering that the maximum protomer length after nanosurgery was 459 nm, compared with PR7's maximum length of 329 nm, the cryptic sites of extensibility in VWF provide a hidden reservoir of extension of up to 130 nm per protomer. 

**Figure 4:**
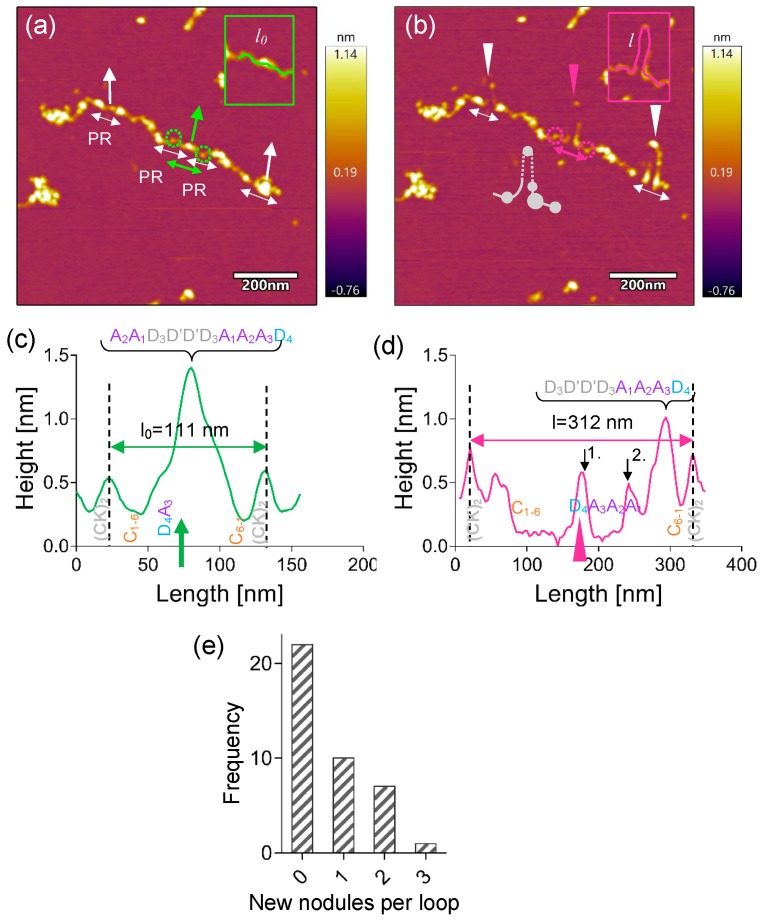
Emergence of small nodules upon VWF nanodissection. (**a**) VWF prior to and (**b**) following nanodissection, in which strand continuity was preserved. The corresponding axial topographical height profiles are shown in (**c**,**d**), respectively. Green and pink double-headed arrows indicate the analyzed VWF sections prior to and following nanodissection, respectively. The detailed analysis of the sections indicated with the white arrows is not shown here. The terminal nodules of the protomers that remained in place after nanodissection are encircled. White and green arrows perpendicular to the VWF multimer axis highlight the path of the AFM tip during the nanodissection procedure. White and pink arrowheads in (**b**) point to the small nodules that appeared in the locally extended loops. The grey cartoon in (**b**) illustrates the schematic structure of the VWF strand following nanodissection. The black arrows on the topographical height profile (**d**) point to the small nodules. Abbreviations: *l_0_*, the original length of the section (green line in inset); *l*, the length after nanodissection (pink line in inset). (**e**) The frequency distribution of the number of nodules within the manipulated VWF section that appeared as a result of nanodissection.

The nanodissection of VWF multimers often resulted in the emergence of protein loops that contained a variable number of nodules along the loop contour (Figure 4). In the example shown (Figure 4a), a relatively compact pre-stretched VWF multimer is seen, in which three protomers were independently nanodissected, which resulted in the emergence of one, two and three small nodules in the loops, respectively (Figure 4b). The middle protomer was analyzed in greater detail (Figure 4a,b insets, Figure 4c,d). Prior to nanodissection, a large nodule could be observed between two neighboring (CK)_2_ nodules, which was interpreted as a complex of D_4_A_3_ and A_2_A_1_D_3_D'D'D_3_A_1_A_2_A_3_D_4_ domains (Figure 4c). Upon nanodissecting the protomer in the region of this large nodule, a protein loop emerged that contained two small nodules that corresponded to the D_4_A_3_ domains and the A_1_ domain, respectively, which were interconnected with an unfolded and extended A_2_ domain (Figure 4d). Notably, the A_1_ domain is separated from the D_3_ of the N-junctional large nodule, which supports earlier findings about the presence of a hinge region between them [21]. Altogether, approximately half of the protein loops that emerged as a result of nanodissection contained at least one small nodule. The distribution of the number of small nodules that emerged is shown in Figure 4e.

**Figure 5:**
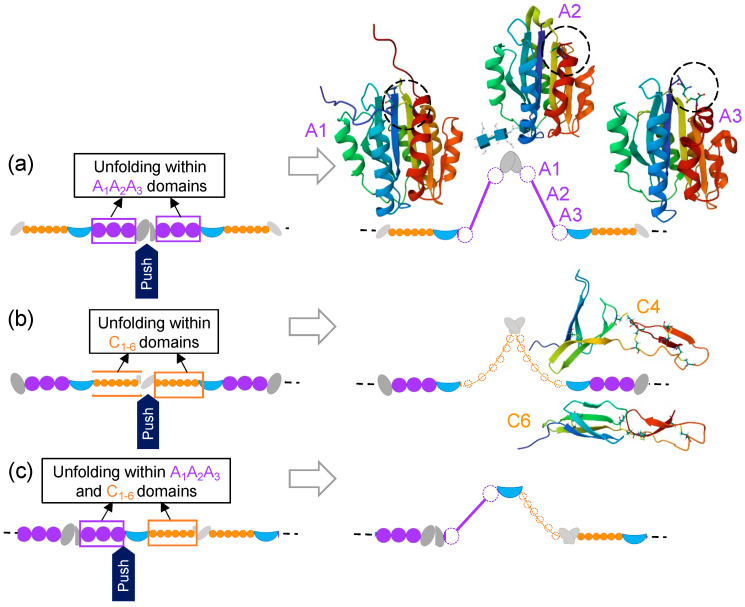
Schematics of the local structural changes that occur by nanodissecting VWF at three distinct locations: (**a**) N-terminal large nodule, (**b**) central (CK)_2_ small nodule and (**c**) A_3_D_4_ small nodule. Domains with similar structure have the same color on the schematic. The three-dimensional structures of the domains that may contribute to elongation were exported from the Protein Data Bank. The chain is colored according to the sequence (blue and red are towards the C-and N-termini, respectively). Disulfide bridges are shown encircled with segmented lines.

In this work, individual pre-stretched, surface-adsorbed VWF multimers were nanodissected by applying mechanical forces locally, at distinct segments and domains of the VWF protomer. Mechanical force may enhance or inhibit chemical reactions, such as protein conformational changes and intra- and intermolecular interactions, depending on the relative orientation of the force with respect to the reaction coordinate [31]. The schematic explanation of how such local forces induced the observed structural changes in VWF is shown in Figure 5. Altogether, regardless of where exactly the point of attack of the mechanical force is, the additional, cryptic extensibility is found in either the A_1–3_ or the C_1–6_ domain regions. Unfolding in these regions results in thinned loop strands, whereas any domain that remains folded appears in the AFM images as small nodules along the loop contour. Although the forces employed during nanodissection are perpendicular to the VWF multimer axis—hence, they are in stark contrast to the forces that VWF is exposed to under in vivo conditions [2,32]—the nanodissection experiments revealed that VWF multimers contain an extensive reservoir of extensibility, which can be liberated under extreme conditions.

## 3. Materials and Methods

### 3.1. Sample Preparation

The source of the VWF multimers was a plasma-derived therapeutic concentrate (Haemate P 1200 IU/500 IU, CSL Behring, Marburg, Germany). VWF was separated into fractions of different molecular weight multimers and from albumin with HiTrap Heparin HP and Desalting columns (GE Healthcare, Chicago, IL, USA) equilibrated with 10 mM Hepes, pH 7.35. Fractions were eluted with 0–0.5 M NaCl gradient and were stored at -80°C until use [5]. Fractions rich in high-molecular-weight multimers were used.

### 3.2. Pre-Stretching VWF Multimers

To pre-stretch the VWF multimers, we used molecular combing, as described previously [5]. Briefly, freshly cleaved mica was mounted horizontally in a custom-built rotor; then, 20 µL of VWF solution (2 µg/mL final in 50% glycerol–PBS pH 7.4; 16 mM Na_2_PO_4_, 4 mM NaHPO_4_, 150 mM NaCl; pH 7.4) was pipetted on the surface. Subsequently, the sample was spun immediately at 13000 rpm (5685 g) for 10 s and then rinsed with MilliQ water for 1 min (Merck Millipore, Burlington, MA, USA) and dried with N_2_ (for 1 min) to obtain a quasi-dry sample. AFM imaging was performed immediately after sample preparation.

### 3.3. AFM Imaging

AFM images were acquired in air with a Cypher S instrument (Oxford Instruments Asylum Research, Santa Barbara, CA, USA) in non-contact (AC) mode by using silicon nitride cantilevers (OMCL-AC160TS-R3, Olympus, Tokyo, Japan, tip radius 7 nm). The setpoint was 60–70% of the free amplitude, and the typical scanning rate was 0.7 Hz. FM images were acquired prior to and following the nanodissection of VWF. 

### 3.4. VWF Nanodissection

To locally dissect the pre-stretched VWF multimer, we carried out nanolithographic procedures on individual strands. The tip of the AFM cantilever was pushed onto the sample and held pressed at a constant force (150–600 nM) and then moved laterally in a pre-set direction (perpendicular to the VWF axis) and with a pre-determined amplitude (maximum 300 nm) and speed (typically 100 nm/s) (steps 1 and 2 in Figure 1d). AFM images were collected with the same cantilever and experimental settings prior to and following nanodissection. The sample temperature (28.3 ± 0.7 °C, mean ± SD) increased by 0.8 ± 0.4 °C during a typical four-hour measurement procedure. 

### 3.5. Image Processing and Data Analysis

Images were processed by using the AR16 software of the AFM, which was based on Igor Pro 6.34 (WaveMetrics, Lake Oswego, OR, USA). Images were corrected for flatness of field and color contrast. The topography was analyzed from section graphs of the manually traced contour of the multimer. The length of the selected section to be manipulated was measured before (*l_0_*) and after the manipulation (*l*) to obtain the loop extension (Δ*l*) as

Δ*l* = *l* − *l*_0_. (1)

In the case of the rupture of the VWF loop, the distance of the gap (*l_r_*) was subtracted to calculate the extension:

Δ*l* = *l* − *l*_r_ − *l*_0_. (2)

The relative extension, *E*, which is the ratio of the final versus the initial lengths, was calculated as

*E* = *l*/*l*_0_, (3)

or, for loops with rupture,

*E* = (*l* − *l*_r_)/*l*_0_. (4)

The topographical height of the sections was measured relative to the background. Further analysis was performed using Microsoft Excel 2016 (Microsoft, Redmond, WA, USA) and Prism 9 (GraphPad, San Diego, CA, USA). Differences between groups were analyzed after the D’Agostino–Pearson normality test by Welch’s *t*-test.

## 4. Conclusions

The nanodissection of pre-stretched, individual VWF multimers revealed that they retain a reservoir of conformational extensibility. Such cryptic extensibility is manifested in the form of thin protein loops that can be pulled out from the VWF backbone. The sites of cryptic extensibility are in the A_1–3_ and C_1–6_ domains, which partially unfold and extend during nanodissection. Cryptic extensibility is considerable, as it may provide up to 3.8-fold relative extension, which corresponds to an additional extension of up to 283 nm per VWF protomer, which is likely to enhance platelet adherence via the exposure of cryptic binding sites. Under extreme mechanical loads, the VWF loops may even become ruptured. The nanodissection procedures employed here may be useful in uncovering the local structural and mechanical features in complex biomolecular systems.

## Figures and Tables

**Table 1:** Length and topographical height parameters of the manipulated sections. The table shows the median values and the interquartile ranges in parentheses.

	Δ*l* [nm]	*E*	*h*_0_ [nm]	Δ*h* [nm]
Continuous VWF loop (*n* = 29)	217 * (182–283)	2.79 * (2.33–3.8)	0.54 (0.45–0.64)	0.28 (0.24–0.38)
Ruptured VWF loop (*n* = 11)	72 * (49–127)	1.57 * (1.27–1.88)	0.58 (0.43–0.62)	0.22 0.20–0.28)

* differences significant between the indicated groups, Δ*l* (*p* = 0.0002) and in *E* (*p* = 0.0001). *h*_0_ is the initial mean topographical height of the section, and Δ*h* is the height drop evoked by nanodissection; Δ*l* is the extension (Equations (1) and (2)), and *E* is the relative extension (Equations (3) and (4)).

## Data Availability

All experimental data, including raw AFM images, are available upon request from the corresponding author.

## References

[1] Bryckaert M., Rosa J.P., Denis C.V., Lenting P.J. (2015). Of von Willebrand factor and platelets. Cell Mol. Life Sci..

[2] Pham O.L., Feher S.E., Nguyen Q.T., Papavassiliou D.V. (2022). Distribution and history of extensional stresses on vWF surrogate molecules in turbulent flow. Sci. Rep..

[3] Lancellotti S., Sacco M., Basso M., De Cristofaro R. (2019). Mechanochemistry of von Willebrand factor. Biomol. Concepts.

[4] Springer T.A. (2014). von Willebrand factor, Jedi knight of the bloodstream. Blood.

[5] Csanyi M.C., Salamon P., Feller T., Bozo T., Harsfalvi J., Kellermayer M.S.Z. (2023). Structural hierarchy of mechanical extensibility in human von Willebrand factor multimers. Protein Sci..

[6] Choi H., Aboulfatova K., Pownall H.J., Cook R., Dong J.F. (2007). Shear-induced disulfide bond formation regulates adhesion activity of von Willebrand factor. J. Biol. Chem..

[7] Zhou Y.F., Eng E.T., Zhu J., Lu C., Walz T., Springer T.A. (2012). Sequence and structure relationships within von Willebrand factor. Blood.

[8] Zhou Y.F., Springer T.A. (2014). Highly reinforced structure of a C-terminal dimerization domain in von Willebrand factor. Blood.

[9] Fowler W.E., Fretto L.J., Hamilton K.K., Erickson H.P., McKee P.A. (1985). Substructure of human von Willebrand factor. J. Clin. Investig..

[10] Bonazza K., Rottensteiner H., Schrenk G., Frank J., Allmaier G., Turecek P.L., Scheiflinger F., Friedbacher G. (2015). Shear-Dependent Interactions of von Willebrand Factor with Factor VIII and Protease ADAMTS 13 Demonstrated at a Single Molecule Level by Atomic Force Microscopy. Anal. Chem..

[11] Löf A., Walker P.U., Sedlak S.M., Gruber S., Obser T., Brehm M.A., Benoit M., Lipfert J. (2019). Multiplexed protein force spectroscopy reveals equilibrium protein folding dynamics and the low-force response of von Willebrand factor. Proc. Natl. Acad. Sci. USA.

[12] Siedlecki C.A., Lestini B.J., Kottke-Marchant K.K., Eppell S.J., Wilson D.L., Marchant R.E. (1996). Shear-dependent changes in the three-dimensional structure of human von Willebrand factor. Blood.

[13] Huizinga E.G., Tsuji S., Romijn R.A., Schiphorst M.E., de Groot P.G., Sixma J.J., Gros P. (2002). Structures of glycoprotein Ibalpha and its complex with von Willebrand factor A1 domain. Science.

[14] Henderson E. (1992). Imaging and nanodissection of individual supercoiled plasmids by atomic force microscopy. Nucleic Acids Res..

[15] Guthold M., Liu W., Stephens B., Lord S.T., Hantgan R.R., Erie D.A., Taylor R.M., Superfine R. (2004). Visualization and mechanical manipulations of individual fibrin fibers suggest that fiber cross section has fractal dimension 1.3. Biophys. J..

[16] Kreplak L., Bar H., Leterrier J.F., Herrmann H., Aebi U. (2005). Exploring the mechanical behavior of single intermediate filaments. J. Mol. Biol..

[17] Wen C.K., Goh M.C. (2006). Fibrous long spacing type collagen fibrils have a hierarchical internal structure. Proteins.

[18] Sziklai D., Sallai J., Papp Z., Kellermayer D., Martonfalvi Z., Pires R.H., Kellermayer M.S.Z. (2022). Nanosurgical Manipulation of Titin and Its M-Complex. Nanomaterials.

[19] Fotiadis D., Scheuring S., Muller S.A., Engel A., Muller D.J. (2002). Imaging and manipulation of biological structures with the AFM. Micron.

[20] Martonfalvi Z., Kellermayer M. (2014). Individual globular domains and domain unfolding visualized in overstretched titin molecules with atomic force microscopy. PLoS ONE.

[21] Zhou Y.F., Eng E.T., Nishida N., Lu C., Walz T., Springer T.A. (2011). A pH-regulated dimeric bouquet in the structure of von Willebrand factor. EMBO J..

[22] Jakobi A.J., Mashaghi A., Tans S.J., Huizinga E.G. (2011). Calcium modulates force sensing by the von Willebrand factor A2 domain. Nat. Commun..

[23] Zhang X., Halvorsen K., Zhang C.Z., Wong W.P., Springer T.A. (2009). Mechanoenzymatic cleavage of the ultralarge vascular protein von Willebrand factor. Science.

[24] Chen P.C., Kutzki F., Mojzisch A., Simon B., Xu E.R., Aponte-Santamaría C., Horny K., Jeffries C., Schneppenheim R., Wilmanns M. (2022). Structure and dynamics of the von Willebrand Factor C6 domain. J. Struct. Biol..

[25] Kutzki F., Butera D., Lay A.J., Maag D., Chiu J., Woon H.-G., Kubař T., Elstner M., Aponte-Santamaría C., Hogg P.J. (2022). Dynamic Disulfide Bond Topologies in von-Willebrand-Factor’s C4-Domain Undermine Platelet Binding. bioRxiv.

[26] Xu E.R., von Bülow S., Chen P.C., Lenting P.J., Kolšek K., Aponte-Santamaría C., Simon B., Foot J., Obser T., Schneppenheim R. (2019). Structure and dynamics of the platelet integrin-binding C4 domain of von Willebrand factor. Blood.

[27] Emsley J., Cruz M., Handin R., Liddington R. (1998). Crystal structure of the von Willebrand Factor A1 domain and implications for the binding of platelet glycoprotein Ib. J Biol Chem.

[28] Buehler M.J., Ackbarow T. (2007). Fracture mechanics of protein materials. Mater. Today.

[29] Beedle A.E.M., Mora M., Davis C.T., Snijders A.P., Stirnemann G., Garcia-Manyes S. (2018). Forcing the reversibility of a mechanochemical reaction. Nat. Commun..

[30] Zhang Q., Zhou Y.F., Zhang C.Z., Zhang X., Lu C., Springer T.A. (2009). Structural specializations of A2, a force-sensing domain in the ultralarge vascular protein von Willebrand factor. Proc. Natl. Acad. Sci. USA.

[31] Webster S.S., Wong G.C.L., O’Toole G.A. (2022). The Power of Touch: Type 4 Pili, the von Willebrand A Domain, and Surface Sensing by Pseudomonas aeruginosa. J. Bacteriol..

[32] Li Y., Xi Y., Wang H., Sun A., Wang L., Deng X., Chen Z., Fan Y. (2023). Development and validation of a mathematical model for evaluating shear-induced damage of von Willebrand factor. Comput. Biol. Med..

